# Persistent Unilateral Facial Inflammation Associated With a Calcium Hydroxylapatite-Compatible Foreign-Body Reaction: A Case Report

**DOI:** 10.7759/cureus.117958

**Published:** 2026-10-15

**Authors:** Macarena Olivares, Nicole Abaroa, Victor Mercado

**Affiliations:** 1 Medicine Academy, Instituto Chileno de Rejuvenecimiento y Optimización de Medicina Estética, Faculdade do Centro Oeste Paulista, Piratininga, BRA; 2 Maxilofacial Ultrasound, Centro Abaroa, High Resolution Maxillofacial Doppler Ultrasound Center, Santiago, CHL; 3 Otolaryngology, Instituto de Neurorehabilitación y Balance, Viña del Mar, CHL

**Keywords:** calcium hydroxylapatite, delayed inflammatory reaction, dermal filler, fibrosis, foreign body reaction, neuropathic pain, ultrasound

## Abstract

Calcium hydroxylapatite (CaHA) is a widely used biostimulatory dermal filler, although persistent inflammatory complications may occur. We report the case of a 23-year-old woman who developed progressive unilateral inflammation of the left midface and lower face several weeks after CaHA injection. Symptoms persisted for approximately two years despite previous diagnoses and treatments. At presentation, she had facial asymmetry, persistent edema, induration, severe pain, and mechanical allodynia. High-resolution ultrasound showed diffuse abnormalities of the subcutaneous adipose tissue, consistent with edema and inflammatory involvement. An incisional biopsy demonstrated fibrous tissue containing exogenous spherules compatible with CaHA and a foreign-body granulomatous reaction. The patient received a multimodal regimen comprising oral and intralesional corticosteroids, pregabalin, collagenase, and lipase. Clinical and ultrasound improvement was observed during ongoing treatment, with residual tissue abnormalities at the latest follow-up. Ultrasound findings did not establish histological resolution of fibrosis. Because interventions were administered sequentially and in combination, improvement cannot be attributed to any single treatment. This case illustrates the complementary roles of clinical assessment, ultrasound, and histopathology in evaluating persistent inflammation after CaHA injection.

## Introduction

Calcium hydroxylapatite (CaHA) is a biodegradable biostimulatory dermal filler composed of synthetic CaHA microspheres suspended in a carboxymethylcellulose gel carrier. It is used for facial volumization and collagen biostimulation because of its biocompatibility and clinical durability [1-4]. Although adverse events are uncommon, delayed inflammatory reactions have been reported with the expanding use of biostimulatory fillers [1-3]. CaHA-related complications include inflammatory nodules, foreign-body granulomas, persistent edema, migration, and fibrosis [1-5]. Their nonspecific clinical presentation may make diagnosis challenging [1,2].

Foreign-body reactions to injectable materials may show granulomatous inflammation and fibrosis, although histological findings vary [3,6]. Diffuse inflammatory involvement of subcutaneous adipose tissue is an uncommon presentation after filler injection [5,7]. Persistent unilateral facial swelling also has a broad differential diagnosis, and the relationship to a prior filler procedure may not be recognized promptly [1,2]. We describe persistent unilateral facial inflammation following a reported CaHA injection, with biopsy findings compatible with a CaHA-related foreign-body granulomatous reaction.

## Case presentation

A 23-year-old woman was referred to our center with a two-year history of persistent swelling involving the left midface and lower face. According to the patient, symptoms began several weeks after facial injection of a CaHA filler reportedly marketed as Hydroxifyll®. The procedure had been performed at another center, and the original treatment records were unavailable. Consequently, the injected volume and concentration, number of sessions, precise injection sites, injection plane, and technique could not be verified.

During the two years before referral, the swelling had been attributed to a presumed temporomandibular disorder. However, no objective evidence confirming temporomandibular joint disease was available, and physiotherapy had not provided satisfactory improvement. At presentation, examination showed marked asymmetry of the left hemiface, diffuse soft-tissue edema, increased facial volume, induration consistent with fibrosis, severe pain, and mechanical allodynia (Figure 1A).

**Figure 1:**
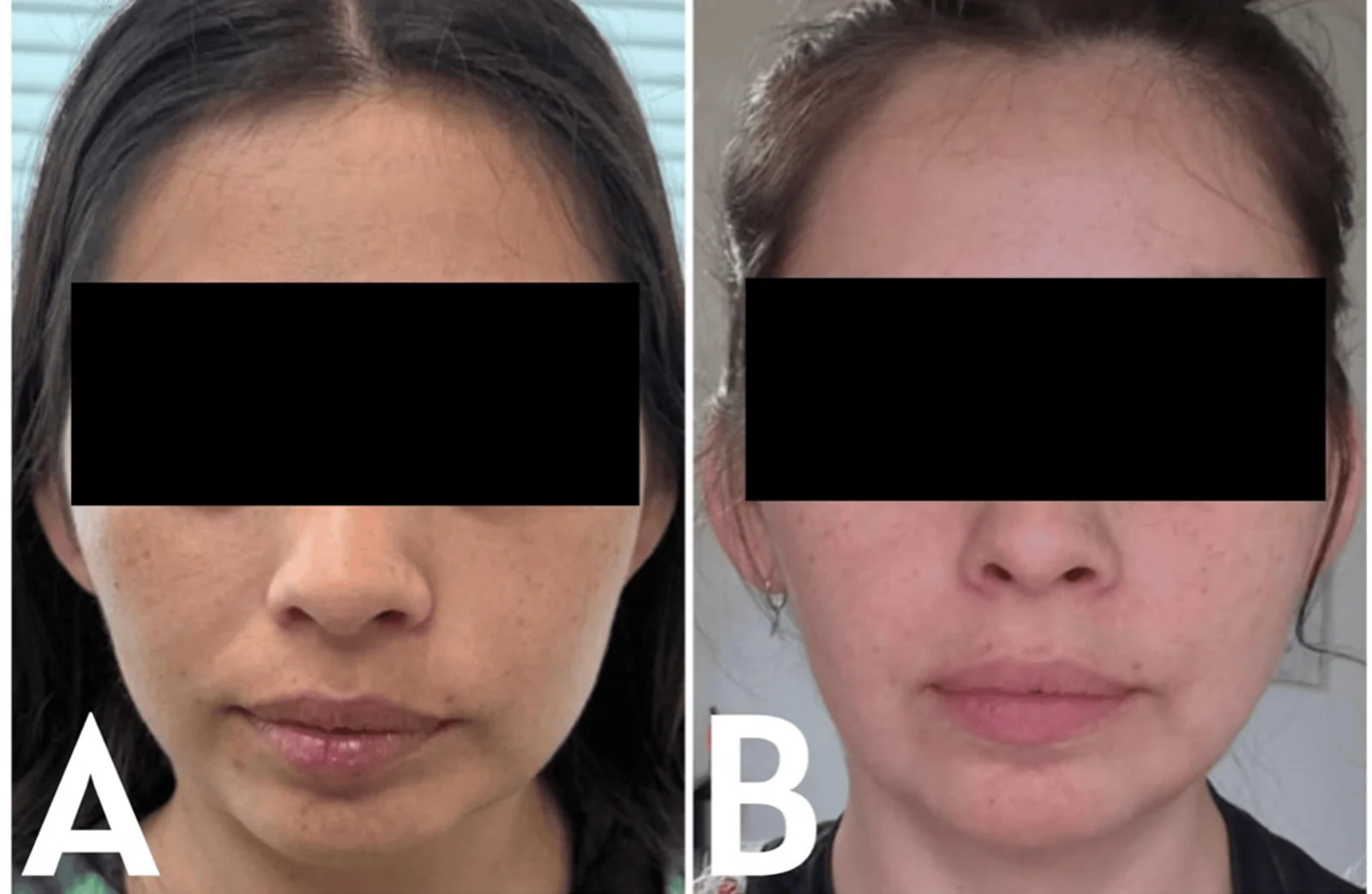
Clinical appearance at presentation and at the latest follow-up during ongoing treatment. (A) Left facial swelling and asymmetry at presentation. (B) Reduced swelling and improved facial symmetry at the latest follow-up. Treatment was ongoing at the time of the follow-up image.

High-resolution ultrasound demonstrated heterogeneous and disorganized echotexture of the subcutaneous adipose tissue, diffuse hypoechoic areas consistent with edema and inflammatory involvement, and reduced definition of the fibrous septa. No organized fluid collection was identified. These findings supported diffuse inflammatory involvement of the subcutaneous tissue and were clinically compatible with unilateral subcutaneous adipose tissue edema (Figure 2).

**Figure 2:**
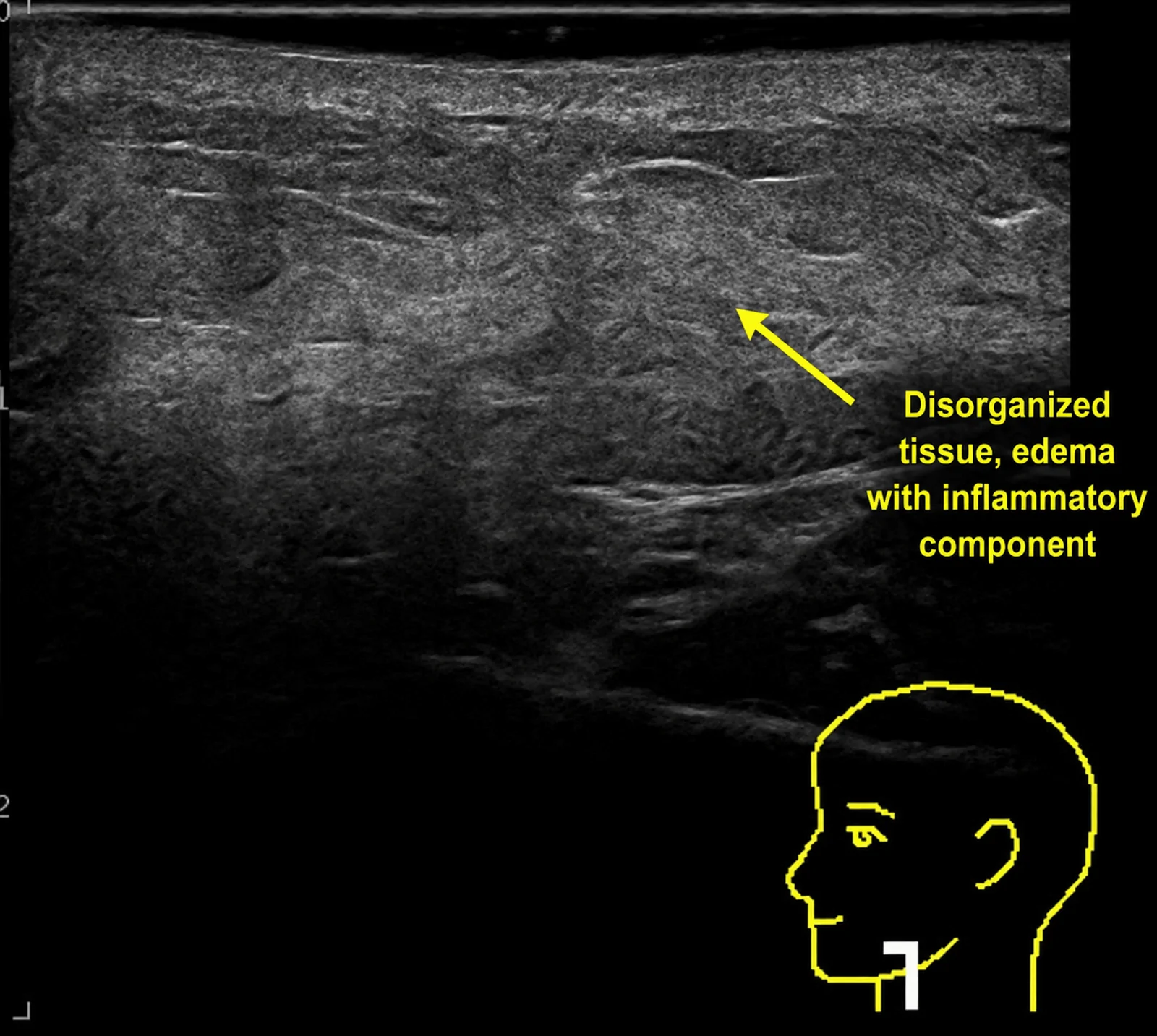
High-resolution ultrasound at presentation. Heterogeneous, disorganized subcutaneous adipose tissue with diffuse hypoechoic areas and reduced definition of the fibrous septa. No organized fluid collection was identified. The yellow side-profile illustration corresponds to the anatomical region where the ultrasound examination was performed. It serves as an anatomical reference to indicate the specific facial area being evaluated.

The chronic course, absence of reported systemic infectious manifestations, and lack of an organized collection on ultrasound made acute infection or abscess less likely. Microbiological cultures, autoimmune studies, and dedicated salivary gland or odontogenic investigations were not available; therefore, these alternative diagnoses could not be fully excluded.

An incisional biopsy of the external cheek was performed. Hematoxylin and eosin-stained sections showed hypocellular, hypovascular fibrous tissue with a tendency toward nodular organization. Two focal areas contained regularly shaped exogenous spherules measuring approximately 30 μm in diameter. The particles had regular borders and were birefringent under polarized light; vacuoles of similar size were also observed. No cellular atypia or malignancy was identified. The histopathological diagnosis was a foreign-body granuloma compatible with CaHA (Figure 3). Histopathology therefore supported a CaHA-compatible foreign-body granulomatous reaction in the context of the reported injection history.

**Figure 3:**
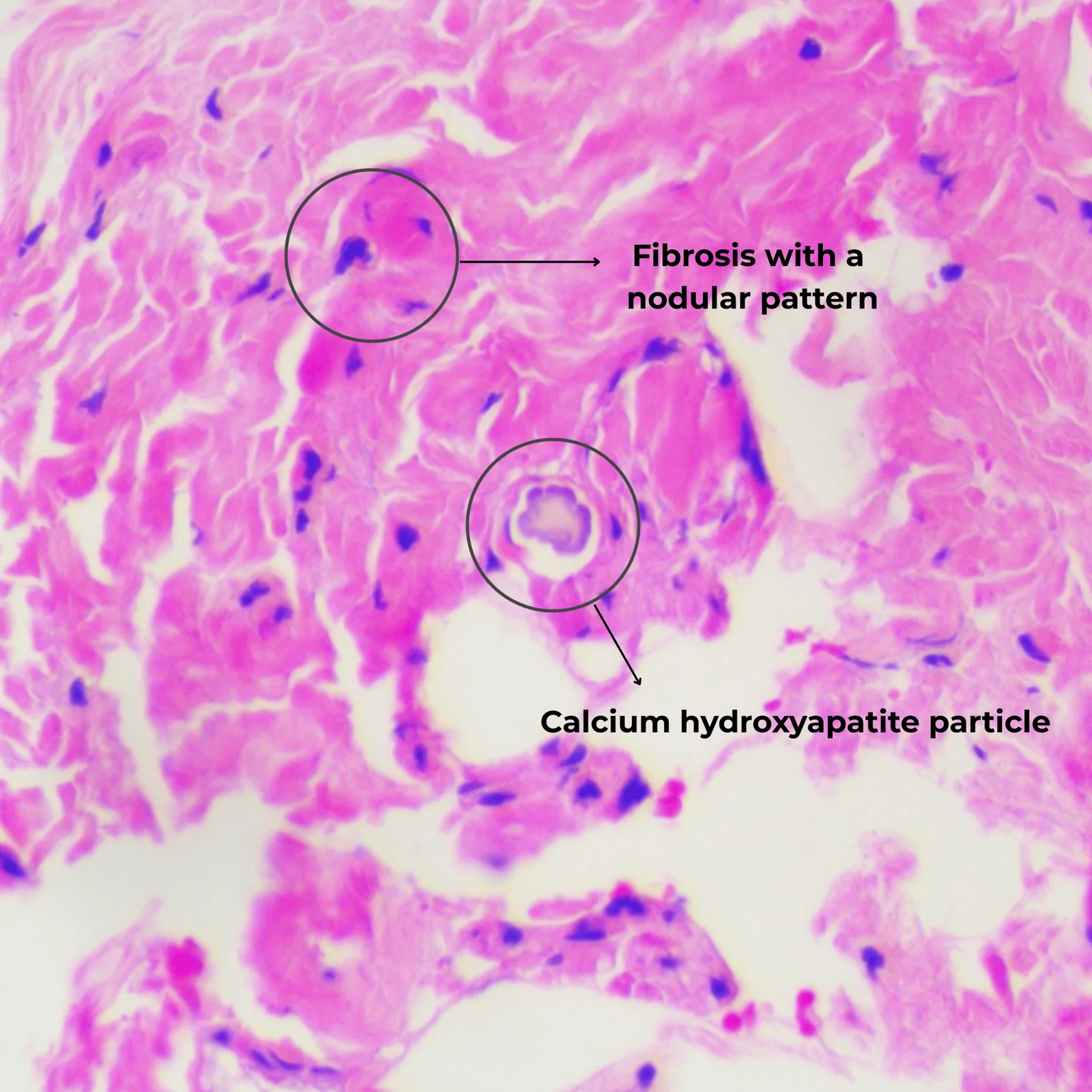
Histopathological findings from the incisional biopsy. Hypocellular, hypovascular fibrous tissue with a tendency toward nodular organization and exogenous spherules with regular borders, compatible with CaHA. The available pathology report did not document whether adequate subcutaneous adipose tissue was sampled.

The available pathology report did not establish whether adequate subcutaneous adipose tissue was sampled. It documented hypocellular, hypovascular fibrous tissue and exogenous spherules, but did not document macrophages or chronic inflammatory infiltrates. Subcutaneous adipose tissue edema remained a clinical diagnosis supported by ultrasound and was not established histopathologically.

The available treatment record indicates that prednisone was started at 20 mg once daily and tapered over 30 days: 20 mg once daily for 10 days, 10 mg once daily for 10 days, 5 mg once daily for 10 days, and then discontinued; this completes a one-month taper. Pregabalin was prescribed at a dose of 75 mg for neuropathic pain for six months. The patient subsequently underwent combined intralesional treatment with collagenase (PBSerum®, Proteos Biotech S.L., Albacete, Spain), lipase (PBSerum®, Proteos Biotech S.L., Albacete, Spain), and dexamethasone sodium phosphate (Biosano, Laboratorio Biosano S.A., Santiago, Chile). Each enzyme preparation contained 1,500 U diluted in 10 mL of saline, yielding a concentration of 150 U/mL. Each session included 1.5 mL of the collagenase preparation and 1.5 mL of the lipase preparation, corresponding to 225 U of each enzyme per session. Dexamethasone sodium phosphate was prepared at 2 mg/mL; 2 mL was administered per session, corresponding to 4 mg. The preparations were distributed throughout the affected hemiface using a 22-gauge cannula. Sessions were performed every 15 days during the first month and monthly thereafter. Treatment remained ongoing at the latest follow-up, approximately one year after initiation. No treatment-related adverse events were recorded in the available clinical documentation.

During treatment, the patient showed progressive improvement in facial edema, induration, asymmetry, pain, and mechanical allodynia. At the latest follow-up, examination showed reduced facial swelling, improved facial symmetry, and better definition of the mandibular contour (Figure 1B). Follow-up ultrasound showed decreased diffuse hypoechogenicity, improved organization of the adipose tissue and septa, and better definition of the fibrous septa. Mild residual tissue heterogeneity and remodeling remained, with no organized fluid collection (Figure 4). Because treatment was still ongoing, the outcome after treatment discontinuation could not be assessed. Because multiple treatments were administered as part of a combined regimen, the observed improvement cannot be attributed to any individual intervention.

**Figure 4:**
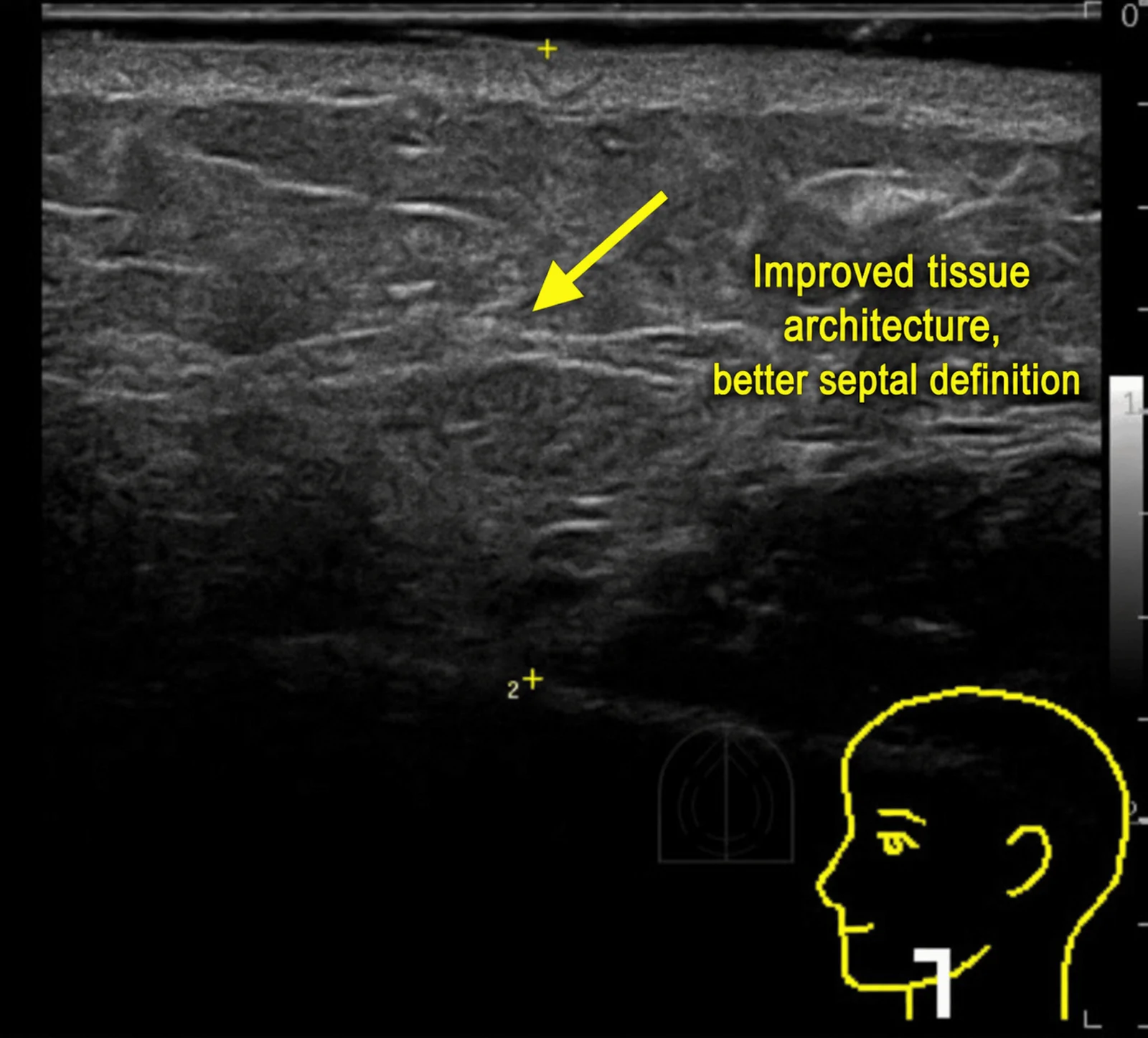
High-resolution ultrasound at the latest follow-up during ongoing treatment. Reduced diffuse hypoechogenicity and improved organization of the adipose tissue and septa, with mild residual tissue heterogeneity and remodeling. No organized fluid collection was identified. The images do not provide comparable quantitative measurements of subcutaneous thickness. The yellow side-profile illustration corresponds to the anatomical region where the ultrasound examination was performed. It serves as an anatomical reference to indicate the specific facial area being evaluated.

Written informed consent was obtained for treatment and for open-access publication of the clinical information and images. Institutional approval was granted under the Faculdade do Centro Oeste Paulista (FACOP) IRB (0078-2026).

## Discussion

In this case, histopathology demonstrated a foreign-body granulomatous reaction compatible with CaHA in the context of the reported injection history. Subcutaneous adipose tissue edema was a clinical diagnosis supported by ultrasound, not a histopathologically established diagnosis. Panniculitis was considered in the differential diagnosis but was not established in this patient.

The interval between injection and symptom onset could only be estimated from the patient’s account because the original procedural records were unavailable. She reported that swelling began several weeks after injection and persisted for approximately two years before the filler-related complication was considered. Thus, the two-year period represents the reported duration of symptoms and diagnostic delay, not the latency between injection and symptom onset.

The patient had no established autoimmune disease but reported a family history of rheumatoid arthritis. No autoimmune investigations were available to evaluate possible immune dysregulation [8]. Implanted biomaterials have been discussed as potential immune adjuvants within the framework of autoimmune/inflammatory syndrome induced by adjuvants (ASIA) [7]. This isolated observation does not establish an association between CaHA injection and ASIA; the concept is mentioned only as a theoretical hypothesis.

Clinical and ultrasound improvement occurred during an ongoing multimodal regimen. Mild residual tissue heterogeneity and remodeling remained at the latest follow-up. Because prednisone, pregabalin, intralesional dexamethasone, collagenase, lipase, physiotherapy, and clinical monitoring formed part of the overall management, improvement cannot be attributed to any one intervention. Treatment had not ended at the latest follow-up, so persistence of improvement after discontinuation could not be assessed.

Pathophysiology

After implantation, CaHA microspheres induce a tissue response involving macrophages, fibroblasts, extracellular matrix remodeling, and collagen deposition [1,3,4,9]. The balance between inflammatory and reparative pathways may influence the subsequent tissue response [1,3]. Persistent stimulation or additional inflammatory triggers may contribute to prolonged macrophage activation and production of inflammatory mediators, including tumor necrosis factor alpha, interleukin 1 beta, and interleukin 6 [1-3,10]. Chronic foreign-body reactions may also involve fibrosis and abnormal extracellular matrix remodeling [3,9].

In this patient, the biopsy documented hypocellular, hypovascular fibrous tissue with nodular organization and exogenous birefringent spherules compatible with CaHA. The available pathology report did not document macrophages or chronic inflammatory infiltrates. The prolonged clinical course may have contributed to the observed induration and fibrotic changes. A relationship between chronic inflammation and mechanical allodynia is biologically plausible through peripheral nerve sensitization, but this mechanism was not directly investigated.

Differential diagnosis

Persistent unilateral facial swelling after cosmetic filler injection presents a broad diagnostic challenge [1-3]. The differential diagnosis includes low-grade or biofilm-associated infection, cellulitis, soft-tissue abscess, impaired lymphatic drainage, salivary gland disease, odontogenic disease, autoimmune panniculitis, vascular abnormalities, and soft-tissue neoplasia [1-3,8].

In this case, the chronic course, absence of reported systemic infectious manifestations, and lack of an organized collection on ultrasound made acute infection or abscess less likely. Histopathology supported a CaHA-compatible foreign-body granulomatous reaction. However, microbiological cultures, autoimmune studies, and dedicated salivary gland and odontogenic investigations were unavailable. Therefore, some alternative causes of unilateral facial swelling could not be fully excluded.

The history of a previous aesthetic procedure was central to interpreting the clinical, ultrasound, and histopathological findings. Inquiry about previous filler injections should be included when evaluating persistent or otherwise unexplained facial swelling.

Role of high-resolution ultrasound

High-resolution ultrasound contributed to the evaluation of the affected subcutaneous tissue after two years of treatment directed toward presumed temporomandibular dysfunction. It showed heterogeneous and disorganized subcutaneous adipose tissue, diffuse hypoechoic areas, reduced definition of the fibrous septa, and no organized fluid collection. These findings supported diffuse subcutaneous tissue involvement. Panniculitis remained a possible differential diagnosis and was not established by ultrasound.

Ultrasound also provided a noninvasive method for qualitative follow-up during treatment. Follow-up imaging showed decreased diffuse hypoechogenicity and improved adipose-septal organization, although mild residual heterogeneity and remodeling persisted. These findings indicate improvement; they do not establish complete normalization or imaging-confirmed resolution of fibrosis.

Ultrasound did not independently establish the definitive diagnosis or exclude every alternative condition. Interpretation required correlation with the clinical history, examination, and biopsy findings. Its accessibility, absence of ionizing radiation, and ability to characterize soft-tissue abnormalities make it a useful complementary examination in patients with persistent facial swelling after filler injection.

Treatment considerations

Standardized protocols for persistent inflammatory and granulomatous reactions associated with CaHA are lacking [1,3]. The available record indicates prednisone 20 mg once daily with a taper over approximately one month; the exact taper schedule was unavailable. Pregabalin 75 mg was prescribed for pain with neuropathic characteristics, but the frequency and duration were not documented. The patient subsequently received combined intralesional treatment consisting of 1.5 mL of collagenase preparation (PBSerum®, Proteos Biotech S.L., Albacete, Spain) and 1.5 mL of lipase preparation (PBSerum®, Proteos Biotech S.L., Albacete, Spain), each prepared by diluting 1,500 U in 10 mL of saline (150 U/mL), corresponding to 225 U of each enzyme per session. Dexamethasone sodium phosphate (Biosano, Laboratorio Biosano S.A., Santiago, Chile) was prepared at 2 mg/mL, and 2 mL was administered per session (4 mg). Sessions were performed every 15 days during the first month and monthly thereafter. Treatment was ongoing at the latest follow-up.

Corticosteroids may reduce inflammatory activity associated with foreign-body reactions [1-3,10], whereas collagenase was included to address associated fibrotic changes [9]. Lipase was used as an adjunct within the combined intralesional regimen [9]. Evidence supporting collagenase and lipase for persistent CaHA-related inflammatory reactions is limited; their individual effects cannot be determined from this case.

Clinical improvement occurred during treatment, including reductions in edema, induration, facial asymmetry, pain, and mechanical allodynia. Ultrasound findings also improved, although residual tissue abnormalities remained. Treatment was ongoing at the latest follow-up; therefore, the outcome and durability after treatment discontinuation are unknown. This case describes the clinical course of a patient managed with a conservative multimodal regimen. It does not establish the efficacy of any specific treatment or support standardized therapeutic recommendations.

## Conclusions

This case describes persistent unilateral facial inflammation temporally associated with a reported CaHA injection. Histopathology supported a CaHA-compatible foreign-body granulomatous reaction, whereas subcutaneous adipose tissue edema remained a clinical diagnosis supported by ultrasound. Panniculitis was considered in the differential diagnosis but was not established. High-resolution ultrasound contributed to assessment and follow-up but did not independently establish the diagnosis or exclude all alternative causes. Clinical and ultrasound improvement was observed during ongoing multimodal treatment, with residual tissue abnormalities at the latest follow-up. Because several interventions were administered sequentially or concurrently, improvement cannot be attributed to any individual treatment. Treatment had not ended; therefore, the outcome and durability after discontinuation remain unknown. This single case cannot establish treatment efficacy or support a standardized management protocol. Further studies are needed to clarify the diagnosis, natural history, and management of similar complications.
